# Obstetrician and gynaecologist engagement in global surgery: adding necessary value to the field

**DOI:** 10.1136/bmjgh-2025-021194

**Published:** 2025-10-27

**Authors:** Parisa N Fallah, Lina Roa, Rohini Dutta, Laurence Bernard, Salome Maswime, Kathleen M Schmeler, Adeline A Boatin

**Affiliations:** 1 Department of Gynecologic Oncology & Reproductive Medicine, The University of Texas MD Anderson Cancer Center, Houston, Texas, USA; 2 Department of Obstetrics & Gynecology, The University of British Columbia Faculty of Medicine, Vancouver, BC, Canada; 3 Department of Obstetrics, Gynecology & Reproductive Sciences, Yale School of Medicine, New Haven, Connecticut, USA; 4 Division of Gynecologic Oncology, Department of Obstetrics & Gynecology, McGill University Faculty of Medicine, Montreal, Quebec, Canada; 5 Division of Global Surgery, Department of Surgery, University of Cape Town Faculty of Health Sciences, Cape Town, Western Cape, South Africa; 6 Department of Obstetrics & Gynecology, Massachusetts General Hospital, Boston, Massachusetts, USA

**Keywords:** Africa, Global Health, Health systems, Surgery, Obstetrics

**SUMMARY BOX**

- Despite growing interest and engagement in global surgery, obstetrician and gynaecologist (OB/GYN) representation remains low, and efforts have focused largely on obstetric care.
- The global burden of gynaecological surgical conditions is large, across a variety of subspecialties in OB/GYN, including benign gynaecology, minimally invasive gynaecological surgery, gynaecological oncology, urogynaecology and family planning.
- Significant effort is needed on the part of the global surgery and OB/GYN communities to unify efforts in addressing this burden.
- Improving access to surgical care for women is critical to reducing morbidity and mortality, improving well-being, and facilitating their ability to thrive as equal members of society.

## Introduction

 In 2015, the Lancet Commission on Global Surgery (LCoGS) demonstrated that 5 billion people lack adequate access to essential surgery, obstetric and anaesthesia care and set clear global targets for 2030 to meet this need.^1^ What followed was a unified commitment to research, advocacy, policy and financial investment for timely access to safe surgical care, focused within low and middle-income countries (LMICs), where the burden of surgical disease is highest. Despite multiple challenges, including the COVID-19 pandemic and minimal funding in LMICs, the infrastructure provided by National Surgical Obstetric and Anesthesia Plans has facilitated some progress towards the 2030 goals.^2^ Ongoing support from a renewed generation of surgical providers is necessary in the coming years.^2^

Although obstetricians and gynaecologists (OB/GYNs) have been involved in global health work for decades, they have not integrated into the nascent field of global surgery.^3^ Thus far, other surgical specialties have largely shaped the global surgery agenda on an international stage, in research, academia, professional meetings, clinical work and capacity-building efforts. On analysing the contributors to the LCoGS report, of the 25 commissioners, only one was a maternal health provider (specifically a nurse-midwife), and there were no OB/GYN surgical providers.^1^ A scoping review of global surgery literature found that only 6% of articles addressed topics within the OB/GYN specialty.^4^ This review suggested that the OB/GYN community is doing significant work within the field of global surgery, but the term ‘global surgery’ itself is not being used when describing that work in the literature, pointing to the further need for a unified approach.^4^

Thus far, the majority of the focus of OB/GYN engagement in global surgery has been on obstetric care. More than 90% of global maternal deaths occur in LMICs and 951 million women live without access to emergency obstetric care.^5^ Moreover, there are increasing concerns regarding the emerging triple burden around caesarean delivery in LMICs: lack of access in some settings, overuse in others and poor quality of surgical care potentially contributing to preventable maternal morbidity and mortality. As such, it is vital that OB/GYN providers are recognised, represented and included in decisions to scale access to safe and affordable surgical care for women.

Furthermore, the work of OB/GYNs extends beyond obstetrics. Women have many reproductive surgical needs apart from their ability to carry a pregnancy and give birth.^3^ A study looking at disparities in the global burden of surgical disease showed that women experience more morbidity from surgically treatable diseases than men, with a ratio of 3:1, specifically due to issues related to their reproductive health.^6^ Despite the significant sex disparity in the burden of global surgical conditions, there is relative inattention within the global surgery field to these conditions, with limited research on levels of access, management and scale-up of care for these conditions.^6^ OB/GYNs are uniquely poised to play a significant role in this space, improving care for women around the world. In addition, OB/GYN represents one of the few specialties that covers a broad spectrum of care, including primary care, labour and delivery, emergency surgery and oncology care. This sweeping focus, ranging from birth to end of life, allows for OB/GYNs to consider surgical needs across the lifespan, with the goal of achieving universal and comprehensive care. Below we describe the multitude of ways in which OB/GYNs contribute to women’s surgical needs across the world and opportunities for collaboration with the global surgery community.

## Benign gynaecological surgery

Benign gynaecological conditions contribute a significant proportion of morbidity for women. This includes acute conditions such as ovarian torsion, ectopic pregnancies and bleeding from miscarriages, as well as chronic conditions such as uterine fibroids, benign ovarian masses, endometriosis, pelvic organ prolapse and incontinence.^7^ While benign gynaecology is not at the forefront of global health efforts, these conditions are associated with debilitating morbidity.

Laparoscopic surgery comprises a large proportion of gynaecological surgery in higher resource settings. Improving access to laparoscopy has the potential to decrease morbidity and mortality from surgery in LMICs, particularly for women. Limited data from LMICs show that less than 1% of hysterectomies are performed laparoscopically and only 10% of all laparoscopic surgeries are done for gynaecological indications, providing a potential area for growth.^7^ This highlights an opportunity for increased engagement of OB/GYNs, particularly minimally invasive gynaecological surgeons, to work with general surgeons to scale up laparoscopic surgery and improve utilisation of existing services in low-resource settings.

## Gynaecological oncology

In the Lancet Oncology Commission report, it was noted that 80% of the 15.2 million people newly diagnosed with cancer each year will require surgical treatment.^8^ It is estimated that by 2030, 45 million cancer patients each year will need surgery, but only 25% of those patients will have access to safe, timely and affordable surgical care.

Within gynaecological oncology, a subspecialty of OB/GYN, cervical cancer is the fourth most prevalent cancer among women worldwide, with over 650 000 new cases and 350 000 deaths each year.^9^ Over 90% of deaths from cervical cancer occur in LMICs, often due to issues with prevention, early diagnosis, screening and treatment programmes, particularly lack of access to high-quality, safe surgery for women with early-stage disease.^9^ In 2020, the World Health Assembly adopted a global strategy for cervical cancer elimination through human papillomavirus (HPV) vaccination, cervical cancer screening and treatment of cervical disease.^9^ In response, increasing attention has been given to HPV vaccination programmes, with countries like Rwanda achieving greater than 90% vaccination coverage.^10^ In addition, cervical cancer screening with HPV testing has come to the forefront of global health efforts. However, less attention has been given to the treatment of pre-cancerous lesions and invasive cancer, with surgical procedures such as loop electrosurgical excision procedure, cold knife cone biopsy, hysterectomy (simple and radical) and pelvic lymph node dissection being critical to those efforts.

Endometrial cancer rates have also been rising at an alarming rate around the world.^11^ Most cases of endometrial cancer can be cured with surgery, including minimally invasive surgery, which is often not available in LMICs.^7^ Similarly, treatment of ovarian cancer requires surgery to optimise outcomes. Building surgical systems to address gynaecological and other cancer needs is critical, and this should happen in conjunction with scale-up of oncologic services such as diagnostics (pathology and imaging), chemotherapy, radiation and palliative care services to meet the significant need in LMICs.

## Fistula repair and urogynaecology

Often a product of poor access to timely caesarean section and obstetric care, obstetric fistula affects 50 000 to 1 00 000 women each year.^12^ Fistula repair, which requires surgical management, can help women reintegrate into society, contribute to their family and local economy, and reduce their risk of gender-based violence. In addition, urogynaecological issues, including pelvic organ prolapse, incontinence and related social and mental health sequelae, represent a significant burden of disease.^12^ It is critical to recognise that access to urogynaecological surgical care can provide significant improvements in quality of life for many women around the world.

Another pressing global issue is female genital mutilation (FGM). The WHO estimates that between 140 and 200 million women and girls have undergone FGM and an additional 3 million are at risk of FGM each year.^13^ The acute and chronic effects of FGM lead to increased maternal morbidity and mortality, as well as detrimental social and mental health effects for the millions of women suffering from its lasting effects. Surgical management of both obstetric fistula and FGM has the potential to address a significant proportion of morbidity for women and requires a comprehensive, culturally sensitive collaboration among public health experts, OB/GYNs, urologists and reconstructive surgeons.^14^

## Family planning and abortion care

On average, 56 million abortions occur each year, of which half are performed in unsafe conditions.^15^ This has led to over 7 million hospital admissions in LMICs annually, as well as maternal death rates between 5% and 13%.^15^ It is necessary to not only address complications of unsafe abortions, but also to provide access to safe abortion care. Manual vacuum aspiration and dilation and curettage are listed on the Disease Control Priorities essential surgeries list, making them an integral part of surgical care for women. Ultimately, achievement of the desired number and healthy timing of births has important benefits for women, families and societies.

## Next steps & challenges ahead

Active engagement of OB/GYN providers in the global surgery community can allow for even greater progress towards both surgical system strengthening and gender equity efforts. In addition to obstetrics, the inclusion of gynaecological surgery is not only critical to ensuring timely access to safe and affordable surgical care, but also in addressing morbidity and mortality for women worldwide. It is important to increase efforts for OB/GYNs to integrate with the global surgery community, particularly in the academic space where international partnerships are formed and high-level policies are developed. In table 1**,** we outline various ways to facilitate this.

**Table 1:** Recommendations for engagement of obstetricians and gynaecologists (OB/GYNs) in current global surgery efforts

Category	Recommendations
Policy: National Surgical Obstetric and Anesthesia Planning	Include OB/GYN leaders from high-income countries (HICs) and low- and middle-income countries (LMICs) in efforts to incorporate maternal and child health plans into emerging National Surgical Obstetric and Anesthesia Plans (NSOAPs) Incorporate efforts to actively address women’s gynaecological and reproductive health needs at a national level into the NSOAP development process
Advocacy: Professional Organizations and Societies	Increase collaboration between national organisations such as the American College of Surgeons (ACS) and American College of Obstetricians and Gynecologists (ACOG) in HICs as well as College of Surgeons of East, Central and Southern Africa (COSECSA) and East, Central and Southern Africa College of Obstetrics and Gynecology (ECSACOG) in LMICs Engage broader international organisations including the International Federation of Gynecology and Obstetrics (FIGO) and the International Society of Surgery (ISS) Use existing models for capacity-building through programmes like the International Gynecologic Cancer Society (IGCS) Global Gynecologic Oncology Fellowship Program Coordinate capacity-building efforts across these organisations to improve efficiency
Academic: Institutional and Clinical Partnerships	Avoid compartmentalising HIC-LMIC institutional collaborations by specialty to prevent duplication of efforts Aim to have cross-specialty engagement within HIC-LMIC institutional collaborations in surgical, OB/GYN and anaesthesia education, resource allocation, and capacity-building efforts Maintain bidirectionality in clinical partnerships between surgery and OB/GYN departments
Research	Include more OB/GYN global health research in surgical journals and use inclusive global surgery terminology within OB/GYN journals Increase partnership between OB/GYNs and surgeons to answer research questions relevant to surgical systems strengthening in both fields
Education	Invite more OB/GYN speakers and participants to surgical meetings Include opportunities to discuss global surgery at general OB/GYN and subspecialty meetings (including gynaecological oncology, minimally invasive gynaecological surgery, and others)

Just as surgery is an ‘indivisible, indispensable part of healthcare’, OB/GYN is a cornerstone of global surgical care.^1^ It is imperative to acknowledge that women deserve equal access to surgical care globally.^3^ The surgical care that OB/GYNs provide ensures that women have the opportunity to live healthy lives, thus improving access to education, their ability to join the workforce, opportunities to contribute to their local and national economies and their right to thrive as equal members of society.

## Data Availability

There are no original data in this work.

## References

[1] Meara JG, Leather AJM, Hagander L (2015). Global Surgery 2030: evidence and solutions for achieving health, welfare, and economic development. Lancet.

[2] Nepogodiev D, Picciochi M, Ademuyiwa A (2025). Surgical health policy 2025-35: strengthening essential services for tomorrow’s needs. Lancet.

[3] Robinson N, Stoffel C, Haider S (2015). Global women’s health is more than maternal health: a review of gynecology care needs in low-resource settings. Obstet Gynecol Surv.

[4] Fowler Z, Dutta R, Kilgallon JL (2022). Academic Output in Global Surgery after the Lancet Commission on Global Surgery: A Scoping Review. World J Surg.

[5] Holmer H, Oyerinde K, Meara JG (2015). The global met need for emergency obstetric care: a systematic review. BJOG.

[6] Powell BL, Luckett R, Bekele A (2020). Sex Disparities in the Global Burden of Surgical Disease. World J Surg.

[7] Schwartz M, Jeng CJ, Chuang LT (2017). Laparoscopic surgery for gynecologic cancer in low- and middle-income countries (LMICs): An area of need. Gynecol Oncol Rep.

[8] Sullivan R, Alatise OI, Anderson BO (2015). Global cancer surgery: delivering safe, affordable, and timely cancer surgery. Lancet Oncol.

[9] Singh D, Vignat J, Lorenzoni V (2023). Global estimates of incidence and mortality of cervical cancer in 2020: a baseline analysis of the WHO Global Cervical Cancer Elimination Initiative. Lancet Glob Health.

[10] Binagwaho A, Wagner CM, Gatera M (2012). Achieving high coverage in Rwanda’s national human papillomavirus vaccination programme. Bull World Health Organ.

[11] Gao S, Wang J, Li Z (2025). Global Trends in Incidence and Mortality Rates of Endometrial Cancer Among Individuals Aged 55 years and Above From 1990 to 2021: An Analysis of the Global Burden of Disease. Int J Womens Health.

[12] Roa L, Caddell L, Ganyaglo G (2020). Toward a complete estimate of physical and psychosocial morbidity from prolonged obstructed labour: a modelling study based on clinician survey. BMJ Glob Health.

[13] (1997). Female genital mutilation: a joint WHO/UNICEF/UNFPA statement. https://apps.who.int/iris/handle/10665/41903.

[14] Foldès P, Cuzin B, Andro A (2012). Reconstructive surgery after female genital mutilation: a prospective cohort study. Lancet.

[15] Grimes DA, Benson J, Singh S (2006). Unsafe abortion: the preventable pandemic. Lancet.

